# Adoption of artificial intelligence in breast imaging: evaluation, ethical constraints and limitations

**DOI:** 10.1038/s41416-021-01333-w

**Published:** 2021-03-26

**Authors:** Sarah E. Hickman, Gabrielle C. Baxter, Fiona J. Gilbert

**Affiliations:** 1 Department of Radiology, University of Cambridge School of Clinical Medicine, Cambridge, UK; 2 Department of Radiology, Addenbrookes Hospital, Cambridge University Hospitals NHS Foundation Trust, Cambridge, UK

**Keywords:** Medical imaging, Medical ethics, Breast cancer, Databases

## Abstract

Retrospective studies have shown artificial intelligence (AI) algorithms can match as well as enhance radiologist’s performance in breast screening. These tools can facilitate tasks not feasible by humans such as the automatic triage of patients and prediction of treatment outcomes. Breast imaging faces growing pressure with the exponential growth in imaging requests and a predicted reduced workforce to provide reports. Solutions to alleviate these pressures are being sought with an increasing interest in the adoption of AI to improve workflow efficiency as well as patient outcomes. Vast quantities of data are needed to test and monitor AI algorithms before and after their incorporation into healthcare systems. Availability of data is currently limited, although strategies are being devised to harness the data that already exists within healthcare institutions. Challenges that underpin the realisation of AI into everyday breast imaging cannot be underestimated and the provision of guidance from national agencies to tackle these challenges, taking into account views from a societal, industrial and healthcare prospective is essential. This review provides background on the evaluation and use of AI in breast imaging in addition to exploring key ethical, technical, legal and regulatory challenges that have been identified so far.

## Background

In breast oncology, a multidisciplinary team approach is essential, with imaging playing a key role in the care pathway for the screening, diagnosis, staging, monitoring and follow-up of malignancies. Novel imaging techniques of increasing complexity have resulted in longer reporting times. This, coupled with a shortage of radiologists and exponential growth in imaging requests, has led to an increasing demand on radiology departments. Recently, there has been a huge interest in using artificial intelligence (AI) for breast imaging to address these pressures, in a speciality where timing is critical and resources are finite.^1^

**Fig. 1:**
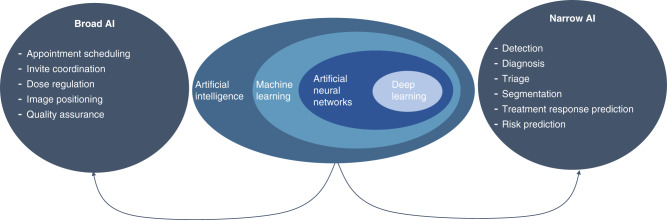
AI applications to breast imaging. The central part of the figure shows the relationship between commonly used terms in the field of AI. The arrows point to the two categories, “Broad AI” and “Narrow AI”, where AI is applied in breast imaging. Examples of these applications are outlined in the lists under each heading.

The term AI covers both machine learning and deep learning.^2^ It is the advances in deep learning for image interpretation that have resulted in the massive growth in interest for use in breast imaging.^3^ AI applications can be broken down into two categories (Fig. 1). The first category is “broad AI”, which lends itself to the administrative and organisational tasks within the imaging pathway. These systems can be used to replace repetitive and routine tasks such as appointment booking, contrast adjustment and image quality checks. The second category is “narrow AI”, which covers computer-aided detection (CADe), diagnosis (CADx), and triaging worklists (CADt) as well as predicting treatment response and segmenting lesions.^3^ These AI systems can be used as aids for clinicians or be used autonomously. Ultimately these AI solutions aim to improve the patient’s outcomes as well as the healthcare system’s efficiency. The latest advances in computer processing and the increased availability of data have been pivotal for developing AI-CAD (CADe and CADx) systems.^4,5^

It is important to remain vigilant to the potential bias and ethical questions that arise when using this technology as well as the challenges of incorporating such systems into pre-existing workflows.^6,7^ These overarching challenges need to be explored in order to facilitate discussion and drive engagement by clinicians, computer scientists, responsible national agencies and National Health Service (NHS) Trusts.^8^

This article reviews how AI has been applied and evaluated using breast imaging as an exemplar. We then consider the ethical and legal challenges at the algorithm, data and clinical levels. Lastly, we discuss the barriers and limitations currently facing this field from a technical, clinical and governance perspective.

## Evaluation of AI in breast imaging

### Retrospective evaluation

Retrospective testing on internal or external datasets is essential when assessing new AI tools for clinical imaging.^4,9^ An algorithm is often trained and tested on an internal dataset which has been divided into an 80:20 split.^9^ This means that the training data is not used to test the algorithm otherwise this would result in bias and an overestimation in performance.^10^ Ideally external datasets consisting of new unseen data which has not been used for algorithm development are used to ascertain the generalisability of an algorithm in different populations with images from different manufacturers (see Ethical and legal constraints—Algorithm level for more information).^9,11^ It is also important to distinguish between testing that is conducted internally (by the AI developers) and externally (by an independent institution). External testing can limit bias and also allow for the comparison of multiple algorithms with similar applications.^12^

Data that is representative of the population, structured, annotated and ready to use is limited, existing in only a small number of institutions (Table 1).^13^ New imaging portals and repositories, such as the Health Data Research Innovation Gateway, have been set-up to try to address these data gaps and are key to developing a data ecosystem to meet the demand.^14^ Principles such as FAIR (findability, accessibility, interoperability, and reusability), aim to guide data extraction as well as long-term management and sharing, in order to obtain the “maximum benefit” from datasets.^15^ However, a balance must be found in this ecosystem between the implementation of FAIR principles and the often-strict controls put in place by Information Governance teams and ethics committees when creating imaging repositories.

**Table 1:** Datasets publicly and privately available for breast imaging.

Dataset	Country	Year of studies	Modality	Number of cases	Number of images
The Mammographic Image Analysis Society Digital Mammogram Database (MIAS)^16^	UK	1994	SF-MG	161	322
Curated Breast Imaging Subset of the Digital Database for Screening Mammography (CBIS-DDSM)^17^	USA	1999 (updated 2016)	SF-MG	1566	10,239
Investigation of Serial Studies to Predict Your Therapeutic Response with Imaging and Molecular Analysis (ISPY1 (ACRIN 6657))^18^	USA	2002–2006	MRI	222	386,528
InBreast^19^	Portugal	2008–2010	FFDM	115	410
Cohort of Screen-Aged Women (CSAW)^20^	Sweden	2008–2015	FFDM	499,807	>2,000,000
The OPTIMAM Mammography Image Database (OMI-DB)^13^	UK	2010–2019	FFDM	151,403	>2,000,000
New York University Breast Cancer Screening Dataset (NYU BCSD v1.0)^21^	USA	2010–2017	FFDM	141,473	1,001,093
Breast Cancer Digital Repository (BCDR)^22^	Portugal	NA	SF-MG FFDM	1010 724	3703 3612
The Cancer Genome Atlas Breast Invasive Carcinoma (TCGA-BRCA)^23^	USA	NA	MRI MG	139	230,167

*FFDM* full-field digital mammography, *MG* mammography, *MRI* magnetic resonance imaging, *NA* not available, *SF* screen film.

The performance of an algorithm can be compared against two outcomes, (1) the ground truth and (2) the radiologist’s performance.^9,10^ The ground truth or “gold standard” is seen as the ‘absolute’ outcome of a case (for example cancer or no cancer) but variations of the ground truth between healthcare systems occur due to differences in standard of care guidelines, histopathology reporting criteria, imaging procedures conducted (e.g. use of magnetic resonance imaging (MRI) versus ultrasound) and screening frequency (e.g. range from 12 to 36 months). The radiologist’s performance sets a “clinically relevant threshold” for AI performance to be compared against and is essential to understand the potential impact of using such systems in real-time workflows (for example double reading in the UK breast screening programme).^11,24,25^ However, in screening when using the radiologist’s assessment as the gold standard, there is potential to introduce bias in favour of the radiologist, where only those patients recalled by the radiologist can be diagnosed by the AI. When trying to prove the superior performance of AI compared with radiologists, interval cancers need to be included in testing sets. Experienced radiologists’ reports should also be included to allow for the comparison against representative programme reader performance, and not just prove that the AI is superior to average or non-specialist performance. Algorithms need to meet or exceed these thresholds in order to show a potential benefit before their adoption into healthcare systems is considered.

### Prospective evaluation

Whilst testing on retrospective datasets provides a “snapshot” of possible performance, the nuances of medical pathways cannot be underestimated. Prospective testing in real-time is essential to fully understand the influence of AI on human performance and the interaction between the two.^4^ There are few prospective studies on the use of AI in radiology (Table 2), with a recent systematic review only reporting one randomised trial registration and two non-randomised prospective studies in radiology.^26^

**Table 2:** Prospective studies for the use of AI in breast imaging.

AI	Country	Imaging modality	Stage of care pathway	Estimated completion	Trial ID (ClinicalTrials.gov)
Samsung (Seoul, South Korea) S-Detect™	China	Ultrasound	Diagnosis	February 2020	NCT03887598
Unknown	China	Mammography	Detection & diagnosis	November 2020	NCT03708978
Unknown	Russia	Mammography (+ others)	Detection	December 2020	NCT04489992
Unknown	China	ABUS	Screening	August 2025	NCT04527510
Kheiron (London, UK) Mia™	UK	Mammography	Screening	Unknown	Unknown—part of the AI Award^7,27^

*ABUS* automated breast ultrasound.

To ensure the clarity of reporting results from these studies, pre-existing reporting standards have been adapted and include the ﻿Consolidated Standards of Reporting Trials-AI (CONSORT-AI), ﻿Standard Protocol Items: Recommendations for Interventional Trials-AI (SPIRIT-AI) and the Checklist for Artificial Intelligence in Medical Imaging (CLAIM).^28–30^ The﻿ Transparent Reporting of a Multivariable Prediction Model for Individual Prognosis or Diagnosis-Machine Learning (TRIPOD-ML) and Standards for Reporting Diagnostic Accuracy Studies–AI (STARD-AI) are also currently under development^31,32^ (Table 3).

**Table 3:** Reporting criteria adapted for AI studies.

	Publication date	Application	Number of items	Link
CONSORT-AI^28^	2020	Randomised trials	25 original 14 new	https://www.equator-network.org/reporting-guidelines/consort-artificial-intelligence/
SPIRIT-AI^29^	2020	Clinical trial protocols	51 original 15 new	https://www.equator-network.org/reporting-guidelines/spirit-artificial-intelligence/
CLAIM^30^	2020	AI studies in radiology	42	https://pubs.rsna.org/doi/full/10.1148/ryai.2020200029
TRIPOD-ML^31^	Pending	Clinical prediction model evaluation	–	https://www.tripod-statement.org
STARD-AI^32^	Pending	Diagnostic accuracy studies	–	–

Performance of AI is often measured in terms of sensitivity, specificity, area under the curve (AUC) and computation time (time taken to process data). Where AI is used by a radiologist, the effect on performance is measured in the same way (sensitivity, specificity, and AUC) with the additional measure of reading time by the radiologist. The AUC provides a summary estimate of diagnostic accuracy, taking into account both the sensitivity and specificity to demonstrate how well the algorithm can differentiate between cancer or not cancer across all thresholds.^33^ It provides a measure between 0 and 1, where a higher score means a better classification.^9^ However, the AUC is subject to certain pitfalls. It is not “intuitive” to interpret clinically, and theoretically algorithms with different sensitivities and specificities can have the same AUC.^33^ Therefore, alternative measures such as “net benefit” have been proposed as well as routine reporting of sensitivity and specificity, which allow for direct clinical comparison.^33^ Lastly, for both the algorithm alone and when used by the clinician, the effect on nationally reported standards (e.g. cancer detection rate, recall rates, tumour size and lymph node status) should be evaluated as part of prospective studies.^9^

### Key considerations for clinical evaluation

Screening AI systems could be cost-effective by improving early detection of important “killer” cancers (higher grade) potentially improving long term survival. However, the substantial investment of AI development, IT infrastructure, and continuous monitoring need to be costed, therefore cost-effectiveness requires careful evaluation.^10,34^ The ease of integrating AI into pre-existing hospital systems, such as radiology information systems and Picture Archiving and Communications Systems (PACS), health-records and administrative systems, is a another key consideration.^25,34^ Wider measures for clinical evaluation to also include are patient acceptability and effect on uptake of screening programmes as well as the training required for radiologists to be able to use and interpret AI tools.^35^

Continuous monitoring to ensure adherence to national standards needs to be in place to observe both static and adaptive (“learning on the fly”) AI when used in real-time workflows (see Ethical and legal constraints—Algorithm level for more information).^25,36^ Each hospital could have an infrastructure to evaluate and monitor algorithms, but this is unlikely to be feasible in many hospitals due to the data storage requirements and lack of technical expertise and resources to set up such an environment. A centralised testing system at designated centres using pre-set national standard thresholds for different AI algorithm applications would be a more sustainable approach.

As outlined above, the steps in the evaluation pathway of AI are clear, requiring retrospective, prospective and continuous real-time testing. However, the caveats of testing such as how to access suitable datasets and defining “clinically relevant thresholds” still need to be agreed. In the UK NHSX has set-up “AI Labs” to begin conducting centralised and standardised testing procedures.^37,38^

## The breast imaging pathway and AI

### Screening

AI has been used in radiology since the 1990’s with initial CADe tools in mammographic screening prompting readers to look again at areas of concern in the image.^39^ More recent AI systems can now meet and exceed the performance of radiologists for stand-alone cancer detection in screening mammography, achieving a sensitivity from 0.562 to 0.819 with a specificity of 0.843–0.966 (set at first reader specificity).^5,12^ However, this is not the case for all national screening programmes.^40^ In a retrospective international crowdsource competition, the performance of multiple algorithms was compared on a standardised test set from Sweden. An ensemble algorithm was built by concatenating the eight best individual performing algorithms, which was shown to outperform the top single algorithm, but not the clinicians performance.^40^

In the UK 2.2 million mammograms are taken each year and read by two radiologists, putting a high demand on an already stretched workforce.^1,35^ The majority of screening mammograms are normal.^35,41^ A more efficient method is sought whilst maintaining current cancer detection and recall standards. AI can now reliably triage “normal” mammograms (47–60%), which would mean that these would not need to be reviewed by two or possibly even one radiologist.^42,43^ Whilst estimated to only miss up to 7% of cancers, the CADt algorithms could drastically improve the efficiency of breast screening. However, questions remain around what an acceptable miss rate would be for algorithms when used in routine screening.

AI tools previously used for mammography have been adapted for other screen imaging techniques such as digital breast tomosynthesis (DBT), which has longer reading times that can be decreased by around 50% using AI.^44^ MRI is used for the screening of high-risk women, particularly those with a familial risk of breast cancer or BRCA1/BRCA2 carriers. Deep learning algorithms can find visual patterns in images and have been used to detect and diagnose breast cancer to produce a fully automated MRI AI-CAD system.^45–47^

### Risk stratification

Screening can be tailored according to a woman’s breast cancer risk. Risk factors for developing breast cancer include breast density, family history, lifestyle factors (e.g., alcohol and smoking), genetic mutations, hormone exposure and expression.^48,49^ Breast cancers can also go undetected due to dense breast tissue obscuring the view of a cancer on a mammogram, called “masking”.^50^ AI density measures can provide quantitative scores or category scores such as BI-RADS, which can provide a more consistent interpretation than a radiologist.^50,51^ It may be possible for the latest density tools to detect women who are at the highest risk of "masking" and more likely to develop a cancer that could progress to later-stage disease.^50^ Automated breast density can also be incorporated into existing prediction models (BOADICEA and Tyrer-Cuzick) to improve performance and assist in the implementation of targeted screening as well as the use of supplemental imaging.^51^ The “measurement challenge” aims to compare automated density measures which have been shown to overcome the inconsistencies in human reporting as well as being able to predict breast cancer risk.^52^

### Monitoring and prognostication

MRI is routinely used in the monitoring of response to neoadjuvant chemotherapy, with patients imaged before, during, and after treatment. Deep learning algorithms have been implemented to evaluate pathological complete response to chemotherapy using post-treatment MRI with an AUC of 0.98,^53^ which could affect the extent of post-treatment surgery, or potentially reduce the need for surgical excision at all. A number of studies have used deep learning to identify features from pre-treatment MRI that are predictive of response in an unsupervised fashion.^54–56^ Early prediction of response to different types of chemotherapy could avoid unnecessary toxicity and cost from ineffective treatment as well as enable a more personalised approach to treatment. AI has also been used in prognostication to predict recurrence (Oncotype DX recurrence score) from MRI.^57^ However, given the moderate accuracy of these techniques (0.77–0.93), further work is required before their integration into clinical practice.

The evidence base for the performance and possible applications of AI to breast imaging is rapidly evolving. Systems acting as stand-alone readers show promise in decreasing workload, whilst systems to predict treatment response could guide tailored treatment strategies. In addition, systems to identify those at greatest risk of a cancer being missed or developing cancer may aid in the application of a targeted screening approach.

## Ethical and legal constraints

### Guidance level

The Department for Health and Social Care, and international collaborations such as the Global Partnership on Artificial Intelligence, have developed guidance for implementing digital technology including AI.^58^ They highlight the need for oversight and continued patient involvement to guide the development of “human-centric” AI which is essential to maintain the trust of the public, and avoid a repeat of previous controversies such as inappropriate data sharing.^59–61^

### Algorithm level

There is a danger of innate latent bias built into certain systems, especially if these have been developed on datasets that underrepresent certain populations (with a lack of diversity in age, ethnicity and socioeconomic background) and therefore lack the ability to generalise.^62^ This could be further compounded by the limited diversity within the scientific workforce itself which under represents the “interests and needs of the population as a whole”.^63^ Outcomes based on pre-existing inequalities could be exacerbated by the skewed outcome being fed back into the algorithm, creating negative reinforcement, thus limiting the fairness of an algorithm.^62^ This can lead to algorithmic decisions that amplify discrimination and health inequalities. The data used in testing should therefore encompass a representative relevant population and the components of the dataset used explicitly reported alongside the results. A recent paper provides an example of such documentation, where an AI-CAD mammography algorithm trained on data from South Korea, USA and UK primarily using data from GE machines, achieved the best performance compared with other algorithms (sensitivity (81.9%) at the reader specificity (96.6%)), when tested on data from Sweden on only Hologic machines, demonstrating generalisability.^12^ Algorithms also have the ability to “learn on the fly”, that over time become more biased due to “performance drift”, thus potentially limiting their generalisability.^36,63^ “Learning on the fly” could potentially be beneficial to adjust algorithms to the local systems in which they are being used but this will also require close observation through regular audits to monitor for detrimental “performance drift”.^10,25^

Transparency around how an algorithm reaches a decision, its architecture and source code availability is often limited by intellectual property clauses to protect proprietary information.^38^ The opaqueness of an algorithm’s deduction can be clarified by using saliency maps, which highlight (e.g. heatmap) the part of the image which the algorithm has used to make its decision, ensuring that the algorithm is using at the correct part of the image to make its clinical deduction and not “noise” in the image such as a clip, artefact or label.^64^ Initial checks built into the algorithm, ensuring the image is of sufficient technical quality from which to deduce an interpretation similar to the checks performed by radiologists, is also an important step for robust interpretation. A reliable algorithm providing consistent, clear and reproducible results, so as not to cause ambiguity in decision making, is key to improving confidence in these systems.

### Who controls the data?

In the UK there is an understanding that NHS Trusts will govern, control and use patient data in an anonymised format to conduct research for patient benefit.^6,65^ There is also an understanding that patient data will be protected and overseen by Information Governance teams at NHS Trusts.^7,37^

Extracting data from the fragmented silos of the NHS remains a challenging task due to the lack of interoperability between systems.^66^ Data relating to an individual’s health is defined as “special category” data and requires additional procedures and safeguards including data minimisation, proportionality, and necessity.^67–69^ Data from which an individual can be recognised is termed personal identifiable data (PID). This data is often pseudonymised or de-identified for healthcare research to remove identifiers and replace them with a new random identifier (e.g., Trial ID), ensuring privacy is upheld.^70^

Where consent from individuals for data use cannot be feasibly obtained, provisions are in place to obtain access to PID in order to create large datasets.^71^ Regulation has emphasised the importance of patient and public involvement (PPI) when using patient data for research, especially in the context of unconsented data use.^71^ Feedback provided by PPI can be used to enhance the communication between the public and healthcare sector, particularly around the distribution of a data notification and objection mechanism.^38,71^ Studies carried out by organisations such as the Wellcome Trust show that the public acknowledge a lack of understanding and hesitancy regarding the uses of health data, particularly when data is shared with and accessed by commercial companies.^72^ National data opt-outs, proposed as part of the Caldicott Review (2016), give patients the option for their data to not be processed.^73^ Recently, the National Data Guardian opened a consultation to revisit the seven Caldicott principles that guide the use of PID and to ensure that public “expectations” should be considered when using confidential information.^74^ However, additional steps need to be taken to inform and educate the public around data use in healthcare so they can be empowered to explore these options.

The expected economic trade-off within the NHS in terms of financial payment, shareholding position or fees for product procurement should be outlined as part of a national policies. Allowing for the potential benefits from sharing valuable NHS data when collaborating with the commercial sector to be realised.^10,58^ It is important to ensure this benefit is fairly distributed across the whole of the NHS to avoid widening gaps in available resources at different Trusts.^8,66^

Linked data across multiple fields such as imaging, genetic and clinical records are of increasing importance for the development of risk prediction models for both prognosis and treatment response. Higher accuracy has been achieved by algorithms when multiple data types are used in training to provide “rich” risk factor information.^75^ Conversely, an understanding of how much data is too much data is required. For example, linking genetics, demographics, home monitoring, smart watch data may mean data is no longer de-identified. In addition, it must be understood that even data collected in large quantities may still be unrepresentative due to a the lack of access to healthcare and ability to participate in research for different populations.^63^

Data provenance, whilst currently not at the forefront of discussions, could become an increasingly tangled web to unwind. Individual Trust data that is currently being used for training algorithms could at the same time be incorporated into the development of centralised evaluation datasets, resulting in a concealed overlap. The ability to track data back to the source and see all of its uses since it left the source via a flag-based system is needed. However, such systems do not currently exist and would not be easy to integrate, let alone to apply to data which has already been processed.

### Clinical level

Clinical acumen must not be lost. AI and clinicians must work in tandem so that if one system fails (e.g. AI) the safety-net of the other system (e.g. radiologists) is in place to avoid harm. However, when AI systems operate alongside clinicians there is a possibility of the clinician becoming over dependent and automation bias to occur.^8,62^ In addition, radiologists might become distracted by prompts from AI, increasing reading time and potentially adversely affecting reader performance.^76^

Where these systems are designed to act independently, human supervision via “pit-stop” analysis of a select cohort of patients, in an audit like fashion, is essential in order to maintain patient safety. The logging and reporting of errors is a potential area of AI automation where human oversight required for the monitoring of AI will necessitate vast amounts of time and resources. Nonetheless in time automation might replace certain aspects of entire jobs. This is juxtaposed against the creation of jobs in the field of healthcare informatics, to create datasets and facilitate the incorporation of AI into hospitals.^38,60^ A potential overarching benefit from automation could be that more time is freed up for clinician interaction with patients and interventions such as image-guided biopsies.

A broader question exists around notifying patients when AI is used in making diagnostic and treatment decisions. Will a patient feel worse if a cancer is missed by an AI tool compared with a human reader? Another consideration is that in certain healthcare systems the prediction of cancer risk could impact patient insurance policies as well as patient mental health by causing anxiety. Therefore, prior to calculations such as the risk of developing a disease, should the patient have to approve this analysis following counselling by a healthcare professional, similar to procedures currently provided for genetic testing?

Overall, these ethical and legal dilemmas should not be underestimated and the provision of guidance from national agencies to tackle these, taking into account views from patients, commercial companies and clinicians, is essential.

## Practical challenges and limitations

### Technical level

Whilst the NHS has state-of-the-art scanners and treatments, it is also still reliant on certain record systems that are paper-based. Thus, technological advancement is a pivotal challenge facing the NHS to allow for the integration of new technology and the flexibility for exporting data on a mass scale.^77^ Modifications to IT capabilities and digitisation of records is vital and should allow for communication and coordination between Trusts.^77,78^ The NHS is also a tightly sealed system; however, companies will need access to update and modify their algorithms. Conversely, caution is needed when opening up systems due increasing the vulnerability to “cyber-attacks”.^79^ How this external access is overseen and governed is a current technical and logistical challenge.

While the majority of data processing within the NHS at present occurs onsite, ‘big data’ processing for image analysis requires the procurement of graphical processing units (GPUs) at Trusts or within cloud-based systems, which may entail the processing of data offsite.^77^ In addition, capacity for larger data storage is needed for the curation of datasets and the storage of additional image analysis provided by algorithms. A lack of clarity still exists around suitable environments and encryption for data storage as well as the level of de-identification required. When de-identifying imaging data it is necessary to retain data that is essential for image viewing, such as the private Digital Imaging and Communications in Medicine (DICOM) tags, whilst ensuring all PID is removed.^80^ As imaging becomes more advanced it is important to ensure that patients cannot be re-identified via the possibilities of image reconstruction, such as reconstructing facial features from computer tomography (CT) or MRI head scans.

### Clinical level

A new multidisciplinary team will need to be developed and trained including clinical scientists and informaticians to work with clinicians to incorporate AI analysis into care decisions.^6,81^ Advancing and generating new technical expertise will require access to training programmes and retention of highly skilled staff who currently re-locate to industry.^38,82^ Programmes such as the NHS Digital Academy are designed to upskill healthcare professionals in areas of digital health as well as leadership and management as part of a national learning programme.^6,81^ The training of radiologists is also set to change with the recent incorporation of AI into the national curriculum.^83^ An openness from commercial companies to disclose the limitations of their algorithms and training radiologists how to interpret these is vital.^8,63^ The use of AI itself to train radiologists or even provide continuous performance monitoring of radiologists are possibilities that need further exploration. Conversely, whether the adoption of such technology will require radiologists to reach a higher level of performance to keep ahead of AI, is subject to ongoing speculation.

### Governance level

Worldwide healthcare systems are moving forward at great pace to try utilising this technology with national funding efforts to develop an AI healthcare ‘ecosystem’. In the UK, this has been facilitated by collaborations from the Accelerated Access Collaborative and NHSX with the formation of the NHS AI labs.^37,38^ The same two bodies have also partnered with the National Institute for Health Research (NIHR) for the provision of an AI Award, to spur investment into promising commercial companies.^27^

The recently published NHSX “buyer’s guide” provides a much needed resource for Trusts when procuring AI technology.^10^ A proposed checklist also published alongside the buyer’s guide gives Trusts a procedure to help ensure vital steps of due diligence are taken, such as setting up insurance cover. However, the overall cost benefit of implementing such systems is limited in its evidence base and more robust evidence is needed to ensure systems are cost-effective.

The legal accountability of algorithms has been at the forefront of healthcare professionals’ questions, as no clear guidance has been produced.^58^ Discussions around the use of AI alongside a radiologist point towards the ultimate responsibility lying with the clinicians, but no specifics have been detailed as to how this would fit with NHS indemnity.^7,8^ For both clinical decision support systems working alongside the radiologist and independent stand-alone systems, further guidance as to the accountability of the companies who developed the algorithm and NHS Trusts using the AI is needed. Reviews of “accidents” and “near misses” arising from the use of AI should be included in department discrepancy meetings. How this is then fed back to companies, to facilitate algorithm improvement, needs to be thought through before such events occur.

## Conclusion

There are many steps to be taken by an array of national agencies, professional bodies and individual NHS Trusts before AI will become common place in breast oncological imaging to help mitigate the growing pressures facing radiology. Whilst promise is shown with algorithms across a range of imaging modalities reaching and in certain cases exceeding human performance, and even performing tasks not feasible for an individual, independent prospective testing against national benchmarks is needed.

Technical integration and upskilling the healthcare workforce is essential for AI adoption. The different ethical and legal dilemmas at the algorithm, data and clinical level should continue to be discussed and guidance updated for healthcare professionals to follow. Further research is needed not only to understand the health economic implications and testing required to ensure that systems are working by meeting the required performance thresholds, but also that latent bias is avoided. Lastly, the legal accountability should be clearly stated for companies and healthcare professionals when using such systems.

## Data Availability

Not applicable.
